# Comparing the clinical effectiveness of different new-born hearing screening strategies. A decision analysis

**DOI:** 10.1186/1471-2458-5-12

**Published:** 2005-01-31

**Authors:** Eva Grill, Franz Hessel, Uwe Siebert, Petra Schnell-Inderst, Silke Kunze, Andreas Nickisch, Jürgen Wasem

**Affiliations:** 1 Department of Physical Medicine and Rehabilitation, University of Munich, Munich, Germany; 2 Institute for Health Care Management, University of Duisburg-Essen, Germany; 3 Institute for Technology Assessment and Department of Radiology, Massachusetts General Hospital, Harvard Medical School, Boston, MA, USA; 4 Program on HTA and Decision Sciences, Institute for Medical Informatics, Biometry, and Epidemiology, University of Munich, Munich, Germany; 5 Institute for Social Paediatrics, University of Munich, Munich, Germany

## Abstract

**Background:**

Children with congenital hearing impairment benefit from early detection and treatment. At present, no model exists which explicitly quantifies the effectiveness of universal newborn hearing screening (UNHS) versus other programme alternatives in terms of early diagnosis. It has yet to be considered whether early diagnosis (within the first few months) of hearing impairment is of importance with regard to the further development of the child compared with effects resulting from a later diagnosis. The objective was to systematically compare two screening strategies for the early detection of new-born hearing disorders, UNHS and risk factor screening, with no systematic screening regarding their influence on early diagnosis.

**Methods:**

**Design: **Clinical effectiveness analysis using a Markov Model.

**Data Sources: **Systematic literature review, empirical data survey, and expert opinion. **Target Population: **All newborn babies.

**Time scale: **6, 12 and 120 months.

**Perspective: **Health care system.

**Compared Strategies: **UNHS, Risk factor screening (RS), no systematic screening (NS). **Outcome Measures: **Quality weighted detected child months (QCM).

**Results:**

UNHS detected 644 QCM up until the age of 6 months (72,2%). RS detected 393 child months (44,1%) and no systematic screening 152 child months (17,0%). UNHS detected 74,3% and 86,7% weighted child months at 12 and 120 months, RS 48,4% and 73,3%, NS 23,7% and 60,6%. At the age of 6 months UNHS identified approximately 75% of all children born with hearing impairment, RS 50% and NS 25%. At the time of screening UNHS marked 10% of screened healthy children for further testing (false positives), RS 2%. UNHS demonstrated higher effectiveness even under a wide range of relevant parameters. The model was insensitive to test parameters within the assumed range but results varied along the prevalence of hearing impairment.

**Conclusion:**

We have shown that UNHS is able to detect hearing impairment at an earlier age and more accurately than selective RS. Further research should be carried out to establish the effects of hearing loss on the quality of life of an individual, its influence on school performance and career achievement and the differences made by early fitting of a hearing aid on these factors.

## Background

Approximately one to three per 1000 children are born with at least moderate, bilateral hearing disorders [1-4]. Children with congenital hearing impairment benefit from early detection and treatment of their hearing loss [5]. The neurological development of hearing abilities requires acoustic stimulation in the first 18 months of life. Deficits due to lack of acoustic stimulation within the first two years are not or not easily recovered by later rehabilitation. The consequences include delayed development of speech and other cognitive and social functions. This delay is already measurable in the first 3 years of life [6]. If disorders are detected and treated in time, either by the use of a hearing device or cochlea implant, most of the children develop normally and do not need additional speech therapy [7,8]. Early diagnosis and treatment within the sensitive time frames are therefore essential. The German consensus conference on neonatal hearing screening proposed diagnosis in the first 3 months and the start of treatment in the first 6 months of life.

Various tests and test combinations with acceptable sensitivity and specificity are available. Transient evoked oto-acoustic emissions (TEOAE) or brainstem evoked responses (BERA) can be measured. One common strategy consists of a two-step TEOAE with the first measurement within the first days of life and a second test a few days later [9] (chapter 2.2.7).

While modern screening techniques for early detection are available there is still a gap between the consensus on detection as early as possible and the current situation. Only 50% to 60% of children with permanent hearing impairment are diagnosed before their second birthday with traditional health care services [2]. While congenital hearing loss is a serious health problem, there is little evidence to support the use of routine universal screening because of the following factors:

• as the prevalence is very low, the positive predictive value of the tests is low

• screening technologies are still in development

• possible costs and consequences are not sufficiently known

• benefits of early intervention are frequently expressed in qualitative terms without presenting unbiased measures of outcome.

In 1995 the US Preventive Services Task Force found insufficient evidence in favour of universal neonatal hearing screening (UNHS) [10]. The Task Force proposed selective screening of new-borns with risk factors to improve the predictive value of the test. In the UK a national neonatal hearing screening programme aimed at detecting bilateral moderate to severe hearing impairments has been recommended [11] and partially implemented within a pilot project [12]. Several studies have modelled the outcomes of UNHS versus risk factor screening [13-15]. Keren et al [16] presented a cost-effectiveness analysis based on a decision tree model which reported short-term effectiveness of UNHS compared to risk factor screening.

However, there is presently no model explicitly quantifying the effectiveness of UNHS versus other programme alternatives in terms of early diagnosis, nor has it been taken into account that diagnosis of hearing impairment within the first few months of life is more "valuable" for the child's further development than diagnosis later in life. Children identified within the developmentally sensitive time frame should therefore be given more weight compared to children with delayed diagnosis.

The objective of this clinical decision analysis was to systematically compare two screening strategies for the early detection of new-born hearing disorders: UNHS and risk factor screening; with the option of no systematic screening regarding their influence on early diagnosis.

Our specific objectives were to show differences between strategies expressed as the number of quality weighed detected child months (QCM), the number of true positive cases at the age of 6 and 12 months, and the number of false positive cases. We also wanted to investigate which parameters had the most influence on the reported differences and how likely these differences were.

## Methods

We developed a clinical decision model to compare two different screening strategies with the option of no systematic screening. Parameters were extracted from the literature, empirically derived from a representative patient survey, and estimated by experts. Univariate and multivariate sensitivity analyses were performed on all relevant parameters.

The decision model was used to predict absolute and incremental effectiveness of two new-born hearing strategies compared with the option of no screening in new-born infants. For the modelling of effectiveness the recommendations of the Panel on Cost-Effectiveness in Health and Medicine were followed [17].

Three possible strategies for neonatal hearing screening (NHS) were evaluated:

- Universal neonatal hearing screening (UNHS): Every hospital-born baby is screened during the first days of life.

- Risk factor screening (RS): The prevalence of hearing disorders is estimated to be higher at risk groups such as children with a positive family history, with congenital infections, cranofacial abnormalities, low APGAR-Score or low birth weight. This strategy screens all children with one or more risk factors for hearing disorders.

- No systematic screening (NS): Children undergo the usual distraction test when presented to the paediatric service during the routine visit at the age of 12 weeks. Some neonates are screened in the hospital, but not in a systematic way. This reflects the present situation in Germany. UNHS is performed in some maternity hospitals and outpatient paediatric services with pilot screening projects in several regions.

The target population of this analysis was all newborn infants. Health effects are expressed as quality weighed number of detected child months (QCM), and as true positive and false positive cases at certain developmentally important ages (6 and 12 months); for example, if a hearing impairment was diagnosed briefly after birth, the infant contributed six QCM at the age of six months. If the infant's hearing loss was diagnosed at the age of five months, the infant added only one detected child month at the age of six months. The term 'quality' is to reflect the idea that the early detection of impairment is a better and desired outcome, although there is no data on quality of life gained by this early detection. QCM, true positives and false positives are reported at the age of 6 and 12 months and with a time horizon of 120 months. Child months which were added up until the age of 6 months were multiplied with a weight of 1, child months added after the age of 6 months were multiplied with decreasing weighting. The derivation of this weight index is described in the section 'data and assumptions' below. We assumed that all children with hearing impairment would be detected before the age of 72 months, the age of school entry, regardless of the kind of screening strategy. In order to give outcomes in the present more weighting, compared to outcomes in the future, future effects have been discounted at an annual rate of 3%. Discounting reflects the higher value of money spent now as opposed to in the future. Similarly, discounting also weights outcomes experienced now (e.g., being diagnosed as true positive) more heavily than those experienced in the future. Table 1 gives the model parameters and their references.

**Table 1:** Model parameters

Parameter	Base case (in %)	Range for sensitivity analysis	Source
Prevalence of new-born hearing impairment	0.15	0.09–0.3	[3], [28], [29], [4], [30], [31], [25]
Prevalence of one or more risk factors for hearing impairment	20	-	[2, 25, 32]
Prevalence of hearing impairment		-	
In children with risk factors	0.38	-	Author's calculation, [33]
In children without risk factors	0.09	-	Author's calculation
Prevalence of risk factors in children with hearing impairment	50	48–56	[1, 28, 34]
Sensitivity of screening	96	96–100	[11, 32, 35]
Specificity of screening	89	77–96	[11, 32, 35]
Sensitivity of diagnostic testing	98	-	
Specificity of diagnostic testing	98	-	
Coverage of screening	90	85–95	Author's estimate
Follow-up after screening	80	75–85	Author's estimate
Healthy children under suspicion of hearing impairment	0.1	-	Author's estimate
Discounting factor	3 per year	0–5	
Weighs for quality adjustment			
Time to diagnosis ≤ 6 months of age	1	-	Experts'estimate
Time to diagnosis > 12 months of age	0.875		
Time to diagnosis > 6 months and ≤ 12 months	linear extrapolation		
Probability of "natural" discovery without systematic screening	Weibull Distribution Median age at diagnosis 18 months	-	Empirical data *)

*) author's calculations, derived from a representative survey, covering all diagnosed cases and the age of diagnosis in Upper Bavaria in 1998 and 1999 [20]

### The decision model

**Figure 1:**
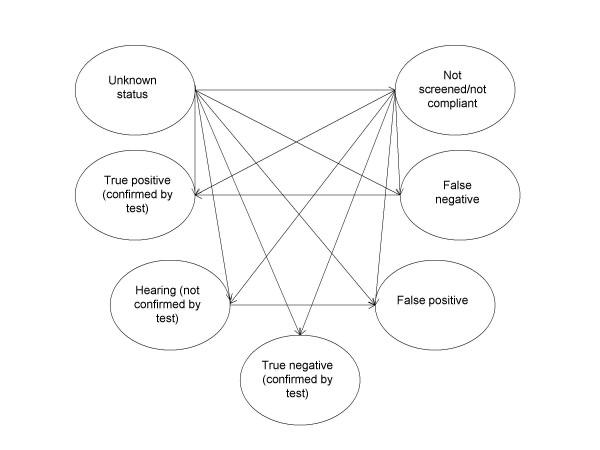
**Health states framework of the Markov model. Arrows indicate the possible transitions. **"Unknown status" is the initial state, "True Positive" and "True Negative" are final (absorbing) states.

We developed a state-transition (Markov) model [18] with monthly cycles to reflect the course of disease and diagnosis under the three screening strategies (figure 1). Probabilistic modelling has been performed by Monte Carlo simulations. The following health states were possible:

- Unknown status

- Healthy (hearing) confirmed by diagnostic test and/or screening – true negative

- Healthy (hearing) not confirmed by diagnostic test

- Hearing impaired confirmed by diagnostic test and/or screening – true positive

- Thought to be healthy (hearing) but hearing impaired – false negative

- Thought to be hearing impaired but healthy (hearing) – false positive

- Not compliant/not followed up

The baseline cohort consisted of infants with a certain prevalence of hearing impairment but unknown status regarding this disorder. Screened and impaired children are detected with the sensitivity of the screening test, whereas screened and healthy children are classified as healthy with the specificity of the screening test. All children with a positive screening test (i.e., true positives and false positives) undergo a second confirmatory diagnostic test, unless they did not adhere to the screening or did not present for the following tests (lost to follow-up). Impaired children who have not been screened in the first cycle can be diagnosed in the subsequent cycles according to the "natural history" of diagnosis, that is, because they don't develop speech adequately or become apparent during the routine visits to the paediatrician. In each cycle children can move to other health states according to the transition probabilities. QCM are only attributed to impaired children in whom hearing impairment is detected. Ultimately, all impaired children of the model cohort are diagnosed as impaired and all healthy children are either classified as healthy or remain unclassified.

Data Professional (TreeAge Inc., Williamstown, MA) was used to construct and run the Markov model and Excel for Windows (Microsoft Corp.) was used to validate the model and to perform the Monte Carlo simulations.

### Data and assumptions

A pre-defined and externally reviewed literature search was performed on new-born hearing screening using all relevant electronic databases. Search strategy and methods have previously been reported in detail [9]. In brief, we searched 13 medical databases including MEDLINE, EMBASE, Current Contents for published papers and HTA databases for published HTA reviews. Relevant articles were identified by a combined text word and thesaurus search. The references of the retrieved articles were then checked for further relevant articles. We restricted our search to publication dates from 1990 to September 2001. Reviewed publications were scored for study quality according to a standardised questionnaire developed by the German Scientific Working Group Technology Assessment for Health Care [19] and then either included or excluded.

All model parameters are shown in Table 1. A two-step screening strategy of Transient Evoked Oto-Acoustic Emissions (TEOAE) was chosen as the model for screening strategies, as this is one of the most widespread and commonly used technologies [9]chapter 2.2.7. The prevalence of congenital hearing disorders in children with risk factors was calculated using the prevalence of children born with one or more risk factors (20%) and the prevalence of risk factors in children with congenital hearing disorders (50%) using Bayes theorem. The probability of presentation with a falsely suspected hearing disorder in hearing children was estimated by a panel of four clinical experts. The probability of detection at a certain age without screening was calculated from a representative survey, covering all diagnosed cases and the age of diagnosis in Upper Bavaria in 1998 and 1999 [20]. A Weibull function for the probability to diagnosis, was fitted to the empirical data. The slope of the weight function has been previously estimated by experts making the following assumptions: each month detected before the age of 6 months is weighted with 1, assuming that children detected (and treated) within the first 6 months of life can develop normal speech and language abilities. If not detected within the first 12 months, profoundly and severely impaired children will conclude with a weight of 0.85, and moderately impaired children with a weight of 0.90. Presuming that 50% of the children with permanent congenital hearing disorders are moderately impaired, gives a weight of 0.875 for every month which is detected after the first birthday. The weights between 6 and 12 months were extrapolated in a linear fashion.

### Model assumptions

Screening procedures and diagnostic procedures are based on different biological and clinical testing principles. We therefore assumed conditional independence of screening procedures and subsequent diagnostic procedures.

### Sensitivity analysis

In order to investigate the influence of the parameter estimates on the outcome measures, one-way sensitivity analyses were performed on all relevant parameters. Ranges used for sensitivity analyses were derived from literature searches and are shown in table 1. Multi-way probabilistic sensitivity analysis was performed on prevalence, sensitivity, specificity, coverage and follow-up using the Monte Carlo technique with 1,000 trials. Point estimates and 95% confidence limits were obtained by counting the number of trials in which a certain strategy has previously been found to be superior to the other strategies [21]. As we assumed that UNHS will always yield more QCM than RS or NS, we also calculated these confidence limits as a function of the difference between strategy effectiveness. This function results in a curve showing the cumulative relative frequency of trials (vertical axis) yielding a certain difference in QCM (horizontal axis) between two alternative strategies. The relative frequency gives an estimate of the probability of a certain difference in clinical effectiveness. The ranges for probability estimates, derived from the literature, assumed beta distribution.

### Model validation

The decision model was validated on three levels:

(i) Technical validation: The model was tested independently with two different software packages (TreeAge Data Professional and MS Excel). Routine tests (e.g., replacing the Weibull function by a constant, setting screening probability equal for all strategies) yielded the expected results.

(ii) Internal validation: All data used to derive model parameter values, were reproduced exactly by the model (e.g., number of detected children at model end point).

(iii) External validation: The derived values are consistent with external projections and estimates of recently published studies which were not used in our model [16,22]. for this section.

## Results

### Base-case analysis

Table 2 presents the results of the base-case analysis. With the base-case prevalence of 150 per 100,000 in a hypothetical cohort of 100,000 children a maximum of 900 QCM would have been accumulated at the age of 6 months, 1800 at the age of 12 months, 18,000 at the age of 120 months (discounted: 892, 1771, 15503), if all children born with hearing impairment had been discovered at birth. UNHS discovered 644 weighted child months before the age of 6 months (72,2% of the expected value). RS yielded 393 child months (44,1%), no systematic screening 152 child months (17,0%). UNHS yielded 74,3% and 86,7% weighted child months at 12 and 120 months respectively, RS 48,4% and 73,3%, NS 23,7% and 60,6%. At the age of 6 months UNHS identified approximately 75% of all children born with hearing impairment, RS 50% and NS 25%. At the time of screening UNHS marked 10% of screened healthy children for further testing (false positives), RS 2%.

**Table 2:** Results of modelling, base case assumption, for a hypothetical cohort of 100,000 children (QCM discounted at an annual 3%)

	Alternative strategies						Expected value
Outcome	UNHS		RS		NS		
		%		%		%	

QCM at 6 months	644	72.2	393	44.1	152	17.0	892
QCM at 12 months	1315	74.3	858	48.4	420	23.7	1771
QCM at 120 months	13436	86.7	11367	73.3	9394	60.6	15503
							
TP at 6 months	112	74.7	74	49,3	38	25.3	150
Incremental TP at 6 months	38		36		-		
TP at 120 months	150		150		150		150
FP after screening	9885		1973		-		-

UNHS = universal neonatal hearing screening

RS = risk factor screening

NS = no systematic screening

QCM = quality weighed detected child months

TP = true positives

FP = false positives.

### Sensitivity analyses

**Figure 2:**
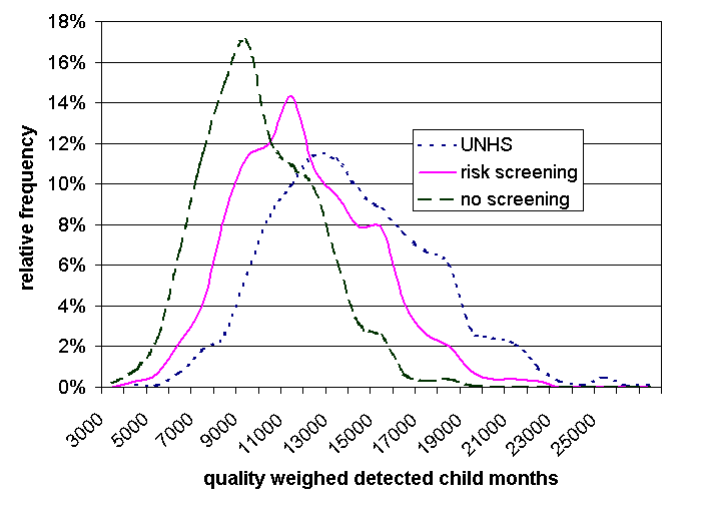
**Distributions of quality weighted detected child months (QCM, 100,000 screened children, 120 months) for newborn hearing screening strategies evaluated by Monte Carlo simulation (UNHS = Universal Newborn Hearing Screening). **For example, if a hearing impairment was diagnosed briefly after birth, the infant contributed six QCM at the age of six months. If the infant's hearing loss was diagnosed at the age of five months, the infant added only one detected child month at the age of six months.

**Figure 3:**
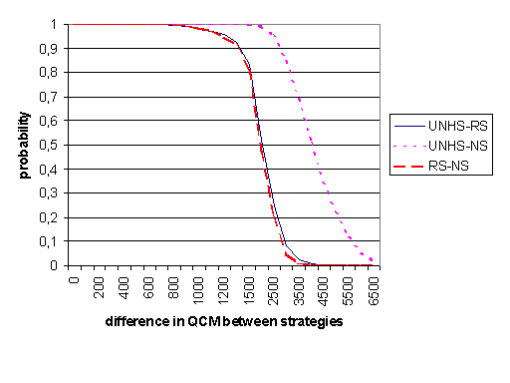
**Probability of a fixed level of incremental quality weighed detected child months (QCM) between strategies. These graphs are the results of a multi-way sensitivity analysis where all model parameters were varied simultaneously within the ranges described in table 1. They give the probability that the incremental gain of QCM between two screening strategies exceeds a certain value (given on the horizontal axis). **This reads as follows: With a probability of 95% the difference between UNHS and RS will be 1200 QCM or more. Results of 1000 trials of a Monte Carlo simulation with a time horizon of 120 months. (UNHS = Universal Newborn Hearing Screening, RS = Risk Screening, NS = No Screening).

Results of sensitivity analyses are presented in table 3. All sensitivity analyses are reported for 120 months follow up. Resulting QCM strongly depended on the prevalence of hearing disorders. Very low prevalence decreased the incremental benefit of UNHS versus RS. Comparing UNHS vs. RS, a prevalence of 9 per 1000 children yielded a gain of 1241 QCM, a prevalence of 15 per 1000 yielded a gain of 2027 QCM. The results were insensitive to varying assumptions about test parameters and the proportion of children lost to follow up. A decrease in slope of the linear weighting function resulted in decreasing incremental QCM. If detected child months were not weighted according to time of diagnosis, UNHS would still be superior to RS and RS to NS in terms of detected child months (data not shown). Figure 2 shows the frequency distributions of QCM per strategy as a result of multi-way probabilistic sensitivity analysis. QCM was higher in UNHS compared to RS or NS in 100% of the 1000 performed trials. Figure 3 shows the cumulative probability of obtaining a given fixed incremental value of QCM. With a probability of 95%, UNHS resulted in a gain of at least 1200 QCM compared with RS and a gain of at least 2500 QCM compared with NS. A gain of 1200 QCM means, for example, that 200 impaired children would be positively diagnosed at birth instead of at the age of 6 months, or that 1200 impaired children would be positively diagnosed before the age of 5 months instead of before the age of 6 months etc.

**Table 3:** One-way sensitivity analyses for QCM at 120 months. The model was evaluated with a range of different values for one parameter while the other parameters were held constant. The ranges of the parameter values are given in table 1

Parameter	Strategy	Lower estimate	Upper estimate
Prevalence			
	UNHS	8061	26872
	RS	6820	22733
	NS	5636	18787
Sensitivity of screening			
	UNHS	13436	13608
	RS	11367	11453
	NS	9394	9394
Specificity of screening			
	UNHS	13436	13436
	RS	11367	11367
	NS	9394	9394
Prevalence of risk factors in impaired children			
	UNHS	13436	13436
	RS	11284	11615
	NS	9394	9394
Coverage of screening			
	UNHS	13158	13714
	RS	11204	11530
	NS	9394	9394
Follow up after screening			
	UNHS	13177	13694
	RS	11237	11496
	NS	9394	9394
Discounting factor			
	UNHS	15725	12124
	RS	13447	10180
	NS	11276	8327

UNHS = universal neonatal hearing screening

RS = risk factor screening

NS = no systematic screening

QCM = quality weighed detected child months

## Discussion

We developed a decision-analytic Markov model for the evaluation of the effectiveness of different new-born hearing screening strategies. In our model, UNHS identified 72% of all detectable QCM at the age of 6 months, RS identified 44% QCM, the control group with no systematic screening identified 17% QCM. UNHS shows higher effectiveness even under a wide range of additional relevant parameters. QCM was introduced as a dynamic time-to-event measure which takes into account that the age of confirmation of congenital hearing impairments is important for further language development. The model results were not sensitive to test the accuracy of parameters within the assumed range but varied with the prevalence of hearing impairment. We have shown that UNHS leads to an earlier age of confirmed diagnosis compared to selective RS.

The cost effectiveness of UNHS has already been modelled along secondary data [16,23,24]. This model is the first to establish a time-dependent and quality-weighted outcome, to introduce empirical data of the natural history of discovery and to present the results within a probabilistic framework. The strength of the presented model is that detected child months are multiplied by a weighted function which adds more benefit per month to children that were diagnosed before 6 months compared to those with late detection. In the existing literature the most interesting outcome, the proportion of children detected early enough, has not been modelled [16].

Our findings are consistent with study results and other projections of effectiveness. The Wessex Universal Neonatal Hearing Screening Trial Group found that 71 more babies with moderate or severe hearing loss per 100 000 target population were diagnosed before the age of 6 months during periods with neonatal screening than during periods without [25]. We found that UNHS would yield 112 true positive cases per 100 000 as compared to 38 without systematic screening, which is a difference of 74 babies. In our model UNHS identified 28% more children in time compared to RS. Thompson et al. estimated this difference to be between 19% and 42% [22] and stated that 77% of hearing impaired children would be identified before 10 months. Keren et al. assumed that UNHS detects 77% and RS 52% of hearing impaired children at the age of 6 months [16]. Any differences may be due to the fact that our function of discovery without systematic screening is more pessimistic.

Our study has several limitations. Firstly, it does not differentiate between bilateral and unilateral hearing loss or moderate and profound impairment. We believe, however, that even with a more refined model the differences in effectiveness would not be substantial. Variations in the degree of impairment would result in variations in sensitivity of the test, and the model was rather insensitive to changes of test parameters. Secondly, even though QCM has been weighted, it is a surrogate parameter for the actual burden of disease for the child. Preference-based utilities have not been measured and the weighting for the impact of early or late identification of hearing loss used in our analysis were estimated by experts. Sensitivity analyses, however, revealed that without weighing, the difference in effectiveness would still be substantial. Thirdly, effectiveness was measured as a function of time to diagnosis. In routine health care, adequate treatment does not necessarily start immediately after the diagnosis is made. However, as knowledge on the consequences of delayed intervention is limited, including time to intervention as another variable in the model would have resulted in decreased precision [22]. Fourthly, our empirical data do not differentiate between congenital and acquired hearing impairment. The rather pessimistic estimation of the detection rate without screening, which might be due to the lack of differentiation between congenital and acquired hearing impairment, may bias the results in favour of UNHS. Similar data, however, have been published ([2,26], presenting a median age at diagnosis 18 months). Our estimate of discovery rate in a setting without screening might be biased by regional differences but can be easily replaced by a constant or a different rate function adapted to other local settings. Fifthly, the impact of late diagnosis on delayed language development, is not yet sufficiently known [22]. The economic effects on society in terms of lost productivity [24] and the quality-of-life effects on individuals have been discussed [27]. There is a lack of evidence concerning health care utilization due to hearing loss and the proportion of children following a regular school and professional career after timely fitting of a hearing aid. Further research should be undertaken to investigate the effect of the age of diagnosis and intervention, on the development of hearing impaired children and on their quality of life.

## Conclusions

The value of this modelling exercise on effectiveness, lies in the facilitation and provision of information to decision makers- by quantitatively projecting available data, making explicit and transparent statements about assumptions and the degree of uncertainty involved in this area. The probability of timely intervention increases with UNHS. UNHS can reduce age of confirmation to a much greater extent than RS. In further studies, our model can be used to predict costs of real life situations to evaluate whether programme implementation costs would surpass cost-effectiveness thresholds. Policy makers can also base their decisions on the incremental effectiveness of UHNS, by introducing a screening programme. The model shows how likely an outcome is under the assumption of parameter uncertainty.

In our model UNHS showed higher clinical effectiveness compared to RS and NS. The strength of our model lies in the naturalistic and generic structure, which makes it useful as a model for further evaluation. The model gives explicit, transparent and quantitative information about the effectiveness of different screening strategies for policy makers who have to decide on the potential impact of neonatal screening. The model presented here is easily adaptable to different settings. It will and should be verified and tested with longitudinal data of ongoing trials and model projects.

This can be one of the first steps towards the needed transparency concerning universal new-born hearing screening.

## Competing interests

The author(s) declare that they have no competing interests.

## Authors' contributions

EG developed the decision model, carried out the statistical analyses and drafted the manuscript. FH designed and coordinated the study and participated in the development of the decision model and in the writing process. US participated in the statistical analyses, in the development of the decision model and in the writing process. PS-I, SK and AN performed the systematic review and data extraction. AN participated in the design of the study. JW conceived of the study and participated in its design and coordination. All authors revised the manuscript and read and approved the final version.

## Pre-publication history

The pre-publication history for this paper can be accessed here:



## References

[B1] Maki-Torkko EM, Lindholm PK, Vayrynen MRH, Leisti JT, Sorri MJ (1998). Epidemiology of moderate to profound hearing impairments in northern Finland: any changes in ten years?. Scand Audiol.

[B2] Fortnum H, Davis A (1997). Epidemiology of permanent childhood hearing impairment in Trent Region. 1985–1993. Br J Audiol.

[B3] Fortnum H, Summerfield AQ, Marshall DH, Davis A, Bamford J (2001). Prevalence of permanent childhood hearing impairment in the United Kingdom and implications for universal neonatal hearing screening: questionnaire base ascertainment study. B M J.

[B4] Parving A, Hauch AM (2001). Permanent childhood hearing impairment – some cross-sectional characteristics from a surveillance program. International Pediatrics.

[B5] Yoshinaga-Itano C, Sedey AL, Coulter DK, Mehl AL (1998). Language of early- and later-identified children with hearing loss. Pediatrics.

[B6] Yoshinaga-Itano C, Apuzzo MRL (1998). The development of deaf and hard of hearing children identified early through the high-risk registry. American Annals of the Deaf.

[B7] Vohr BR, Carty LM, Moore PE, Letourneau K (1998). The Rhode Island Hearing Assessment Program: Experience with statewide hearing screening (1993–1996). J Pediatr.

[B8] Finitzo T, Albright K, O'Neal J (1998). The newborn with hearing loss: Detection in the nursery. Pediatrics.

[B9] (2004). Deutsche Agentur für Health Technology Assessment des Deutschen Instituts für Medizinische Dokumentation und Information; Arbeitsgruppe Health Technology Assessment Neugeborenenhörscreening: Hörscreening für Neugeborene. Hörscreening für Neugeborene : ein Health-Technology-Assessment der medizinischen Effektivität und der ökonomischen Effizienz, Niebüll: Medicombooks.de. http://gripsdb.dimdi.de/de/hta/hta_berichte/hta063_bericht_de.pdf.

[B10] United States Preventive Services Task Force (1996). Guide to Clinical Preventive Services. USPSTF.

[B11] Davis A, Bamford J, Wilson I, Ramkalawan T, Forshaw M, Wright S (1997). A critical review of the role of neonatal hearing screening in the detection of congenital hearing impairment. Health Technol Assess.

[B12] Davis A, Hind S (2003). The newborn hearing screening programme in England. Int J Pediatr Otorhinolaryngol.

[B13] Kemper HC, Downs SM (2000). A cost-effectiveness analysis of newborn hearing screening strategies. Arch Pediatr Adolesc Med.

[B14] Turner RG (1991). Modeling the cost and performance of early identification proocols. J Am Acad Audiol.

[B15] Turner RG (1992). Comparison of four hearing screening protocols. J Am Acad Audiol.

[B16] Keren R, Helfand M, Homer Ch, McPhillips H, Lieu TA (2002). Projected Cost-Effectiveness of Statewide Universal Newborn Hearing Screening. Pediatrics.

[B17] Weinstein MC, Siegel JE, Gold MR, Kamlet MS, Russell LB (1996). Recommendations of the panel on cost-effectiveness in health and medicine. JAMA.

[B18] Sonnenberg FA, Beck JR (1993). Markov models in medical decision making: a practical guide. Med Decis Making.

[B19] Siebert U, Behrend C, Mühlberger N, Wasem J, Greiner W, vd Schulenburg JM, Welte R, Leidl R, Leidl R, von der Schulenburg JM, Wasem J (1999). Development of a Criteria Catalogue for the Description and Assessment of Economic Evaluations in Germany. [German; original title: Entwicklung eines Kriterienkataloges zur Beschreibung und Bewertung ökonomischer Evaluationsstudien in Deutschland]. Ansätze und Methoden der ökonomischen Evaluation – eine internationale Perspektive Health Technology Assessment (Comissioned by the German Agency of Health Technology Assessment at the German Institute for Medical Documentation and Information / Federal Ministry of Health).

[B20] Bornschein B, Grill E, Brockmeier SJ, von Kries R (2003). Diagnosezeitpunkt spracherwerbsrelevanter frühkindlicher Schwerhörigkeit in Südbayern. Informatik, Biometrie und Epidemiologie in Medizin und Biologie.

[B21] Critchfield GC, Willard KE (1986). Probabilistic analysis of decision trees using Monte Carlo simulation. Med Decis Making.

[B22] Thompson DC, McPhillips H, Davis RL, Lieu TA, Homer CJ, Helfand M (2001). Universal newborn hearing screening. Summary of evidence. JAMA.

[B23] Kezirian EJ, White KR, Yueh B, Sullivan SD (2001). Cost and cost-effectiveness of universal screening for hearing loss in newborns. Otolaryngol Head Neck Surg.

[B24] Mohr PE, Feldman JJ, Dunbar JL, McConkey-Robbins A, Niparko JK, Ritterhouse RK, Skinner MW (2000). The societal costs of severe to profound hearing loss in the United States. Int J Technol Assess Health Care.

[B25] Kennedy CR, Kimm L, Dees DC, Campbell MJ, Thornton ARD, Bamber J (1998). Controlled trial of universal neonatal screening for early identification of permanent childhood hearing impairment. Wessex Universal Neonatal Screening Trial Group. Lancet.

[B26] Kiese-Himmel C, Ohlwein S (2000). Die Sprachentwicklung sensorineural hörgestörter Kleinkinder. Sprache Stimme Gehör.

[B27] Davis A, Hind S (1999). The impact of hearing impairment: a global health problem. Int J Pediatr Otolaryngol.

[B28] Gross M, Finckh-Krämer U, Spormann-Lagodzinski M-E (2000). Angeborene Erkrankungen des Hörvermögens bei Kindern. Teil 1. HNO.

[B29] Nekahm D, Weichbold V, Welzl-Mueller K (2001). Epidemiology of permanent childhood hearing impairment in the Tyrol, 1980–94. Scandinavian Audiology.

[B30] Finckh-Krämer U, Spormann-Lagodzinski M-E, Gross M (2000). German registry for hearing loss in children: results after 4 years. Int J Pediatr Otorhinolaryngol.

[B31] Watkin PM, Baldwin M (1999). Confirmation of deafness in infancy. Arch Dis Child.

[B32] Aidan D, Avan P, Bonfils P (1999). Auditory screening in neonates by means of transient evoked otoacoustic emissions: A report of 2,842 recordings. Ann Otol Rhinol Laryngol.

[B33] Vohr BR, Widen JE, Cone-Wesson B, Sininger YS, Gorga MP, Folsom RC (2000). Identification of neonatal hearing impairment: Characteristics of infants in the neonatal intensive care unit and well-baby nursery. Ear and Hearing.

[B34] Fonseca S, Forsyth H, Grigor J, Lowe J, MacKinnon M, Price E (1999). Identification of permanent hearing loss in children: are the targets for outcome measures attainable?. Br J Audiol.

[B35] Baumann U, Schorn K (2001). Früherkennung kindlicher Hörschäden. Visuelle und automatische Verfahren im Vergleich. HNO.

